# Susceptibility of European Red Deer (*Cervus elaphus elaphus*) to Alimentary Challenge with Bovine Spongiform Encephalopathy

**DOI:** 10.1371/journal.pone.0116094

**Published:** 2015-01-23

**Authors:** Mark P. Dagleish, Stuart Martin, Philip Steele, Jeanie Finlayson, Samantha L. Eaton, Sílvia Sisó, Paula Stewart, Natalia Fernández-Borges, Scott Hamilton, Yvonne Pang, Francesca Chianini, Hugh W. Reid, Wilfred Goldmann, Lorenzo González, Joaquín Castilla, Martin Jeffrey

**Affiliations:** 1 Moredun Research Institute, Pentlands Science Park, Bush Loan, Penicuik, Near Edinburgh EH26 0PZ, United Kingdom; 2 Animal Health & Veterinary Laboratories Agency Lasswade, Pentlands Science Park, Bush Loan, Penicuik, Near Edinburgh EH26 0PZ, United Kingdom; 3 Neurobiology Division, The Roslin Institute at, Royal (Dick) School of Veterinary Studies, University of Edinburgh, Easter Bush Campus, Midlothian, EH25 9RG, United Kingdom; 4 CIC bioGUNE, Parque tecnológico de Bizkaia, Derio 48160, Spain; 5 IKERBASQUE, Basque Foundation for Science, Bilbao 48013, Bizkaia, Spain; Colorado State University, College of Veterinary Medicine and Biomedical Sciences, UNITED STATES

## Abstract

European red deer (*Cervus elaphus elaphus*) are susceptible to the agent of bovine spongiform encephalopathy, one of the transmissible spongiform encephalopathies, when challenged intracerebrally but their susceptibility to alimentary challenge, the presumed natural route of transmission, is unknown. To determine this, eighteen deer were challenged via stomach tube with a large dose of the bovine spongiform encephalopathy agent and clinical signs, gross and histological lesions, presence and distribution of abnormal prion protein and the attack rate recorded. Only a single animal developed clinical disease, and this was acute with both neurological and respiratory signs, at 1726 days post challenge although there was significant (27.6%) weight loss in the preceding 141 days. The clinically affected animal had histological lesions of vacuolation in the neuronal perikaryon and neuropil, typical of transmissible spongiform encephalopathies. Abnormal prion protein, the diagnostic marker of transmissible encephalopathies, was primarily restricted to the central and peripheral nervous systems although a very small amount was present in tingible body macrophages in the lymphoid patches of the caecum and colon. Serial protein misfolding cyclical amplification, an *in vitro* ultra-sensitive diagnostic technique, was positive for neurological tissue from the single clinically diseased deer. All other alimentary challenged deer failed to develop clinical disease and were negative for all other investigations. These findings show that transmission of bovine spongiform encephalopathy to European red deer via the alimentary route is possible but the transmission rate is low. Additionally, when deer carcases are subjected to the same regulations that ruminants in Europe with respect to the removal of specified offal from the human food chain, the zoonotic risk of bovine spongiform encephalopathy, the cause of variant Creutzfeldt-Jakob disease, from consumption of venison is probably very low.

## Introduction

Bovine spongiform encephalopathy (BSE) is infectious to, primarily, domestic cattle but also wild bovids, several species of antelope, sheep and felids and is considered to be the cause of variant Creutzfeldt-Jakob disease (vCJD) in humans [1,2]. It is a member of the transmissible spongiform encephalopathies (TSE) group of diseases, also known as prion diseases, which include, amongst others, scrapie in sheep and goats, sporadic, familial and iatrogenic Creutzfeldt-Jakob disease (CJD) and kuru in humans, transmissible mink encephalopathy in ranched mink and chronic wasting disease (CWD) in farmed and free-living cervids [3]. All TSEs are invariably fatal and are characterised by long incubation periods leading to clinical neurological manifestations. The pathology is usually linked to the conversion of the normal host-encoded membrane-bound prion protein (PrP^C^) to the abnormal disease-associated isoform (PrP^d^) which accumulates in the nervous system and, depending on the host species and the TSE agent involved, in the lymphoreticular system [4] and also some other viscera including kidney, muscle and adrenal [5]. Definitive diagnosis of TSEs is, at present, dependent on the detection of abnormal PrP in tissues by immunohistochemistry (PrP^d^) or of proteinase resistant abnormal PrP (PrP^res^) by a variety of biochemical methods [6]. Susceptibility to natural, presumed orally, acquired TSEs is influenced by the age at exposure and the dose of challenge and epidemiological studies in cattle have suggested younger animals are more susceptible to BSE [7]. Additionally, oral BSE challenge of newborn sheep may be more efficient than sheep exposed to oral BSE as adults [8,9]. Similarly, following scrapie exposure it has been shown experimentally, with respect to both attack rate and incubation period, pre-weaned lambs are at greater risk of infection, possibly due to patent gut epithelium [10].

Polymorphisms in the prion protein gene (*PRNP*) determine the degree of susceptibility and resistance to TSEs in several species and also frequently influence the length of the incubation period [11]. Development of clinical disease in classical scrapie and BSE in sheep is primarily associated with polymorphisms in three specific codons (136, 154 and 171) of the ovine *PRNP* gene although other codons may play a role as well [12,13]. No such strong genetic association with susceptibility or resistance to BSE appears to exist for domestic cattle derived from the *Bos taurus* lineage and most seem at risk (reviewed in [12]). However, in British and German cattle herds more complex variations in the regulatory regions of the *PRNP* gene may play a role in the control of susceptibility to BSE [14–17]. In humans, codon 129 is strongly influential for susceptibility to both sporadic and variant forms of CJD [18]. In deer, a total of 16 polymorphic codons within *PRNP* have been reported with a large number of *PRNP* alleles and amino acid substitutions found in white-tailed deer (*Odocoileus virginianus*), European red deer (*Cervus elaphus elaphus*), reindeer (*Rangifer tarandus*) and mule deer (*Odocoileus hemionus*) [19]. A degree of genetically conferred resistance to CWD seems to occur in white-tailed deer due to polymorphisms in codons 95 and 96 [20] and in mule deer due to a polymorphism in codon 225 [21] but in neither case is full resistance conferred. No naturally occurring TSE has been diagnosed in reindeer to date but the *PRNP* sequence suggest they would be susceptible to CWD [22] and recent experimental studies have proven this [23]. With respect to CWD in Rocky mountain elk (wapiti, *Cervus canadensis nelsoni*) heterozygosity (methionine (M)/leucine (L)) in codon 132 of *PRNP*, which is the equivalent position to human *PRNP* codon 129 [24], has been proposed to provide some protection [25] although this has been disputed [26] and other studies suggest this polymorphism primarily alters the length of the incubation period [27].

A *PRNP* polymorphism in codon 226 (glutamine (Q)/glutamate (E)) has been described for European red deer and Sika deer (*Cervus nippon*) [28], but not yet for the Rocky mountain elk, and the association of this amino acid change with TSE susceptibility has recently been demonstrated in transgenic mice [29]. Codon 226 glutamine is encoded in *PRNP* from mule deer [30], white-tailed deer [20], moose (*Alces alces*), cattle, sheep, goats and nyala (*Nyala angasii*) [12] whereas glutamate is encoded in *PRNP* from Rocky mountain elk, kudu (*Tragelaphus* spp.) and cats all of which have shown natural susceptibility to the CWD or BSE agent [12,31,32]. Retrospective analysis showed European red deer of all codon 226 *PRNP* genotypes were susceptible to BSE when challenged by the intra-cerebral route [33] M.P. Dagleish pers observation), which is important as they are consumed by humans so if natural transmission takes place they are a potential source of vCJD. However, their susceptibility to oral challenge, the accepted route of natural BSE transmission, is unknown.

The aim of this study was to assess the susceptibility of European red deer to oral challenge with a bovine derived BSE brain homogenate to assess transmission by the presumed natural route and therefore the potential risk of zoonotic transmission of BSE from red deer.

## Materials and Methods

### Animals, BSE inocula, challenge procedure and biopsies

Twenty-five European red deer were housed at 1–2 days old and hand reared with milk replacer prior to weaning onto *ad libitum* hay and water and a weight dependant allocation of proprietary concentrate feed. At 7–10 weeks of age, 18 animals (10 males and 8 females) were each given 25g of a pool of BSE-positive bovine brain material (VLA-Weybridge SE1736 BBP1) [33] diluted 1:4 (w/v) in 0.32M sucrose solution (total volume 100ml) via a stomach tube followed by flushing of the stomach tube with 50ml of water. Unchallenged environmental controls (n = 7, 3 males and 4 females) were kept in separate but adjacent pens to the challenged deer. Animals were examined daily for clinical signs and weighed at regular intervals. Animals were culled at 190 days post-challenge (dpc) (n = 6 BSE-challenged and 1 unchallenged control), 365 dpc (n = 6 BSE-challenged and 2 unchallenged controls) or allowed to progress until clinical signs or the termination of the experiment at 2320 dpc (n = 6 BSE-challenged and 4 unchallenged controls) (Table 1). Four biopsies were taken from the recto-anal mucosa-associated lymphoid tissue (RAMALT) of the group allowed to progress until clinical signs/termination of the experiment and evaluated for the presence of PrP^d^ by immunohistochemistry (IHC, see below) at 511, 706, 855 and 1853 dpc. Biopsies were performed as described previously [34] with the addition of reversible sedation (10mg/kg medetomidine hydrochloride, Domitor, Janssen Animal Health, UK; atipamezole hydrochloride, Antisedan, Janssen Animal Health, UK). All experimental procedures were approved by the Moredun Research Institute Animals Experiments Ethical Committee and authorised under the UK Animals (Scientific Procedures) Act 1986.

**Table 1:** Summary of animal, immunohistochemical and serial protein misfolding cyclical amplification data.

Deer ID	Sex	Challenge status	DPC-PME	Genotype (PrP gene, codon 226)	IHC for PrP^d^	sPMCA 1^st^ Exp. (3^rd^ round)	sPMCA 2^nd^ Exp. (3^rd^ round)	sPMCA 2^nd^ Exp. (4^rd^ round)
322	M	UNC	190	QQ	N	0/4	0/4	0/4
319	M	UNC	365	QE	N	-	-	-
334	M	UNC	365	EE	N	0/4	0/4	0/4
032	F	UNC	2320	QQ	N	-	-	-
034	F	UNC	2320	QE	N	-	-	-
039	F	UNC	2320	EE	N	1/4	0/4	0/4
014	F	UNC	2320	EE	N	-	-	-
327	M	BSE-C	190	QE	N	-	-	-
309	M	BSE-C	190	EE	N	-	-	-
031	F	BSE-C	190	QQ	N	-	-	-
320	M	BSE-C	190	QE	N	-	-	-
324	M	BSE-C	190	QQ	N	-	-	-
326	M	BSE-C	190	QQ	N	-	-	-
323	M	BSE-C	365	QE	N	0/4	0/4	0/4
337	M	BSE-C	365	EE	N	0/4	0/4	0/4
314	M	BSE-C	365	QQ	N	0/4	0/4	0/4
318	M	BSE-C	365	QE	N	-	-	-
310	M	BSE-C	365	QE	N	-	-	-
028	F	BSE-C	365	QQ	N	0/4	0/4	0/4
009	F	BSE-C	1727	QQ	P	4/4	4/4	4/4
015	F	BSE-C	2320	QE	N	0/4	0/4	0/4
033	F	BSE-C	2320	EE	N	0/4	0/4	0/4
016	F	BSE-C	2320	EE	N	0/4	0/4	0/4
023	F	BSE-C	2320	QE	N	0/4	0/4	0/4
037	F	BSE-C	2320	EE	N	0/4	0/4	0/4

Deer identification, sex, challenge status, time of post-mortem examination, genotype at codon 226 of prion protein gene, presence of abnormal protein by immunohistochemistry (PrP^d^) and western blotting (PrP^res^) by western blotting after different experiments of serial protein misfolding cyclic amplification (sPMCA). The animal positive for abnormal prion protein is deer 009 and this was by both IHC and sPMCA. Note that for sPMCA a value of 1/4 is considered negative and probably due to contamination of the sample in this exceptionally sensitive technique. DPC-PME = days post challenge of post mortem examination, PrP = prion protein, PrP^d^/PrP^res^ = abnormal prion protein, IHC = immunohistochemistry, sPMCA = serial protein misfolding cyclic amplification, Exp. = experiment, M = male, F = female, UNC = unchallenged negative control, BSE-C = BSE oral challenged, Q = glutamine, E = glutamic acid, N = negative, P = positive, for sPMCA x/x = number of PrP^res^ positive tubes/number of replicates, – = not examined by sPMCA.

### Genotyping

Genotyping was performed as described previously [33], either from blood samples taken from live deer into vacutainers containing EDTA (BD Bioscience, Erembodegem, Belgium) or from frozen brain material collected post-mortem. Briefly, PCR amplification and sequencing were performed as described previously [35], using amplification oligonucleotides DeerPrP-213d (AGGTCAACTTTGTCCTTGGAGGAG) and DeerPrP+139u (TAAGCGCCAAGGGTATTAGCAT) and sequencing oligonucleotides DeerPrP+70u (GCTGCAGGTAGATACTCCCTC) and DeerPrP-86d (CAGTCATTCATTATGCTGCAGACT).

### Post-mortem examination

All animals were subjected to full post-mortem examination after euthanasia by intra-venous pentobarbitone after sedation as described above and an extensive range of tissue samples were taken, as described previously [33], and fixed in 10% neutral buffered formalin and/or stored at −80°C. Fixed samples were post-fixed in fresh 10% neutral buffered formalin, processed for histology routinely and then embedded in paraffin wax. Sections (4μm thick) were mounted on glass slides (Superfrost slides, Menzel-Gläser, Braunschweig, Germany) and either stained with haematoxylin and eosin (HE) or subjected to IHC for PrP^d^ (see below).

### Immunohistochemical localisation of PrP^d^

This was performed as described previously [33,36] using two primary antibodies to PrP which have wide inter-species reactivity; F99, clone 97.6.1 (VMRD Inc., Pullman, USA), which binds to amino acid sequence 220–225 of human PrP and BAR224 (CEA, Saclay, France) which recognizes amino acid sequence 141–147 of human PrP. Briefly, antigen retrieval included immersion in 98% formic acid for 5 minutes followed by autoclaving for 30 minutes at 121°C in 0.2% citrate buffer pH6.0. After blocking steps to quench endogenous peroxidase activity and to block reactivity of non-specific tissue antigens, incubation with the primary antibody was carried out overnight at 27°C. The subsequent steps of the immunohistochemical protocol to visualise the bound primary antibodies were performed using a commercial immunoperoxidase technique (Vector-elite ABC kit, goat anti-mouse IgG and DAB chromagen, all Vector Laboratories, Peterborough, UK) at the end of which sections were immersed in 0.5% copper sulphate to enhance immunoperoxidase colour reaction and finally counterstained with Mayer’s haematoxylin.

Ovine BSE shows reduced patterns of intracellular labelling with antibodies that recognise the extreme N-terminus of PrP and that are characteristically different from most scrapie sources [5]. To confirm the BSE nature of the infection, serial sections of selected brain areas were incubated with the N-terminal 12B2 monoclonal antibody (kindly provided by Jan Langeveld, Central Institute for Animal Disease Control, Netherlands), which recognizes amino acid sequence 93–97 of ovine PrP [37], and subjected to the same IHC protocol as described above.

### Protein Misfolding Cyclic Amplification (PMCA)

Serial rounds of PMCA were performed as described previously [38]. Briefly, 50μl of cattle brain homogenate, confirmed to be BSE negative, was supplemented with 0,5% sulfated dextran (Mr ~70,000) from Leuconostoc spp. (Sigma-Aldrich) and seeded with 5μl of red deer brain samples (10% brain homogenate in phosphate buffered saline) from 13 of the animals (n = 6 BSE challenged animals that either developed clinical signs or were culled at the end of the experiment at 2320 dpc, n = 4 culled at 365 dpc and n = 3 environmental controls also culled at the end of the experiment at 2320 dpc). To ensure bovine brain homogenate was a suitable substrate for European red deer samples with respect to serial PMCA (sPMCA) an initial titration (10^−1^ to 10^−11^) was performed using red deer brain material that was known to be positive for PrP^d^ by IHC (deer ID 009, see below).

All 13 red deer brain samples (in quadruplicate) were subjected to two independent experiments of sPMCA. All resultant samples were analysed for PrP^res^ (proteinase resistant PrP which is another disease associated form of PrP^C^) by western blotting using monoclonal antibody 2A11 diluted 1:2000 [39]; for the first experiment after round 3 and for the second experiment after rounds 3 and 4. Unseeded tubes (n = 4) containing only the BSE negative cattle brain homogenate were included as a negative control preparation.

## Results

### Clinical signs

None of the animals subjected to post-mortem examinations at either 190 or 365 dpc showed any clinical signs of disease. Of the six remaining BSE challenged deer, a single animal (ID 009, female) developed acute clinical signs at 1726 dpc. These included fear of people (prior to this the animal was very tame and actively approached people in the pen), restlessness, pacing, stereotypic head movements and low head carriage, abnormal flicking of the ears, laboured and audible mouth breathing and ptyalism. The animal was euthanased the following day (1727 dpc) on welfare grounds and subjected to full post-mortem examination. At post-mortem examination the animal weighed 57.6 Kg and had lost 27.6% (21.9 Kg) of its body weight compared to its peak weight of 79.5 Kg recorded at 1586 dpc. None of the five remaining BSE challenged deer, nor any of the negative control animals, developed clinical signs or weight loss, other than small seasonal variations, by the termination of the experiment at day 2320 dpc.

### Genotype

All animals were genotyped at polymorphic codons 132 and 226 of *PRNP*, other amino acid changes were not deduced from the gene sequences. All deer in the study were homozygous for methionine at codon 132 whereas they were of mixed genotypes at codon 226 (Table 1). The only animal which developed clinical disease was homozygous for glutamine (QQ) at codon 226 and this was the only animal of that genotype in the BSE-challenged group of longest duration which was comprised of six deer allowed to progress to clinical signs or termination of the experiment (Table 1).

### Histological lesions and immunohistochemical labelling of PrP^d^

PrP^d^ was not detected by IHC within any of the RAMALT biopsy samples from any of the deer over the whole course of the experiment.

**Figure 1:**
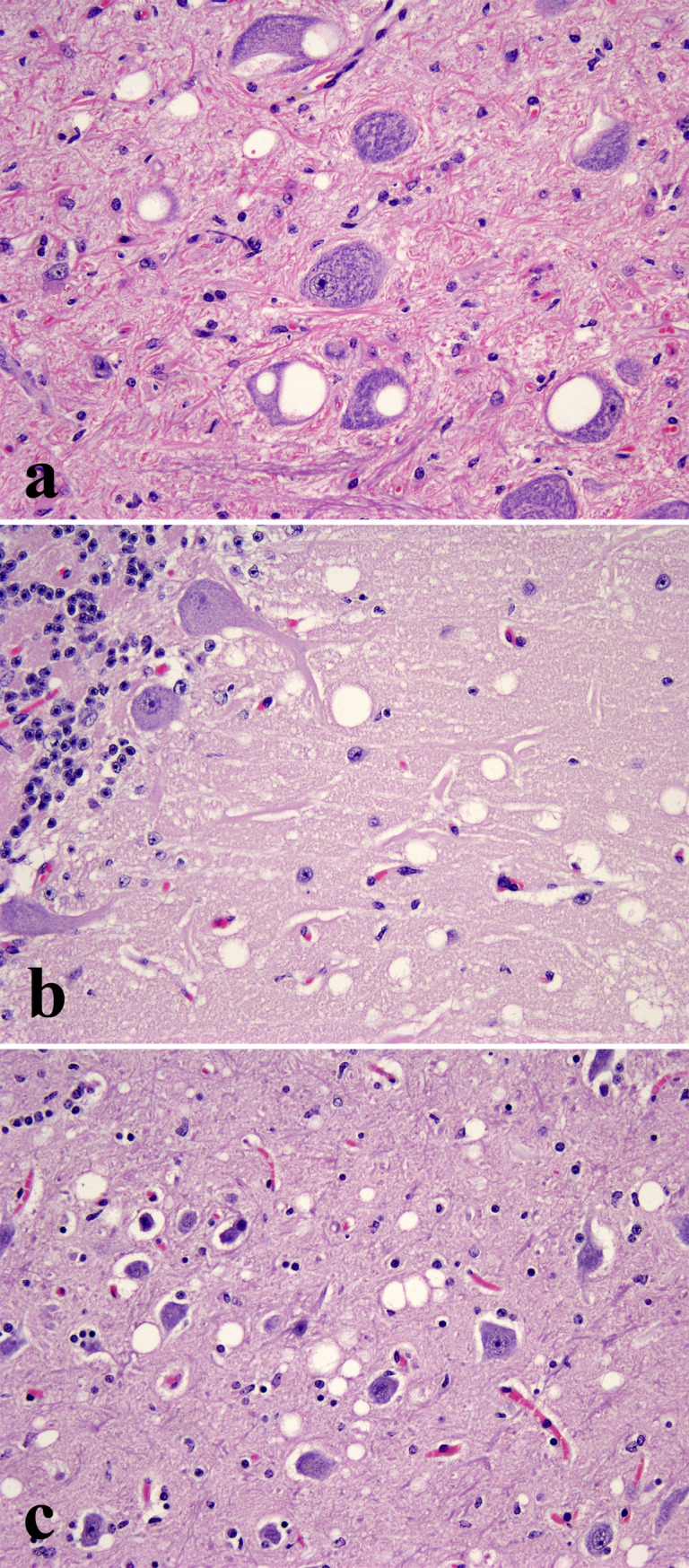
Vacuolation of neuronal perikarya and neuropil in the dorsal motor nucleus of the vagus nerve (DMNV) in the medulla oblongata (a), the molecular layer of the cerebellum (b) and the thalamus (c) of the only clinically affected deer (ID 009). HE, x200.

Microscopical examination of HE stained sections of brain from the single clinically affected deer (ID 009) revealed widespread spongiform change with vacuolation seen in the neuronal perikaryon and neuropil of the brainstem (Fig. 1A), the molecular layer of the cerebellum (Fig. 1B) and thalamus (Fig. 1C). No such lesions were observed in any of the other 24 deer either BSE-challenged or unchallenged controls and no significant lesions were present in any of the other tissues examined.

**Figure 2:**
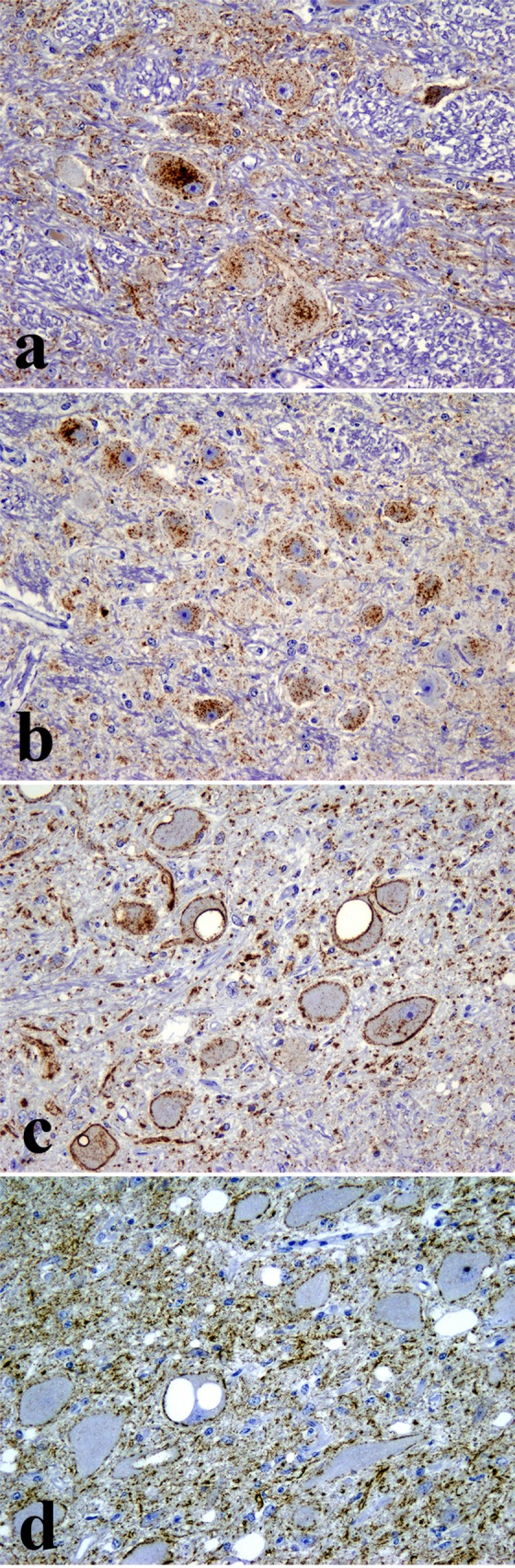
Accumulation of PrP^d^ (brown pigment) in the brain of the only clinically affected deer (ID 009). Diffuse particulate and punctate intraneuronal PrP^d^ in the spinal tract nucleus of the trigeminal nerve (a) and inferior olivary nucleus (b); IHC with antibody F99, clone 97.6.1 x200. (c) Diffuse particulate, peri-neuronal and punctate intraneuronal PrP^d^ in the dorsal motor nucleus of the vagus nerve with antibody F99, clone 97.6.1 (x200). Only the diffuse particulate and peri-neuronal PrP^d^ types remain within the dorsal motor nucleus of the vagus nerve while the punctate intraneuronal PrP^d^ type is unlabelled after incubation with the PrP N-terminal specific 12B2 antibody (d; x200).

**Figure 3:**
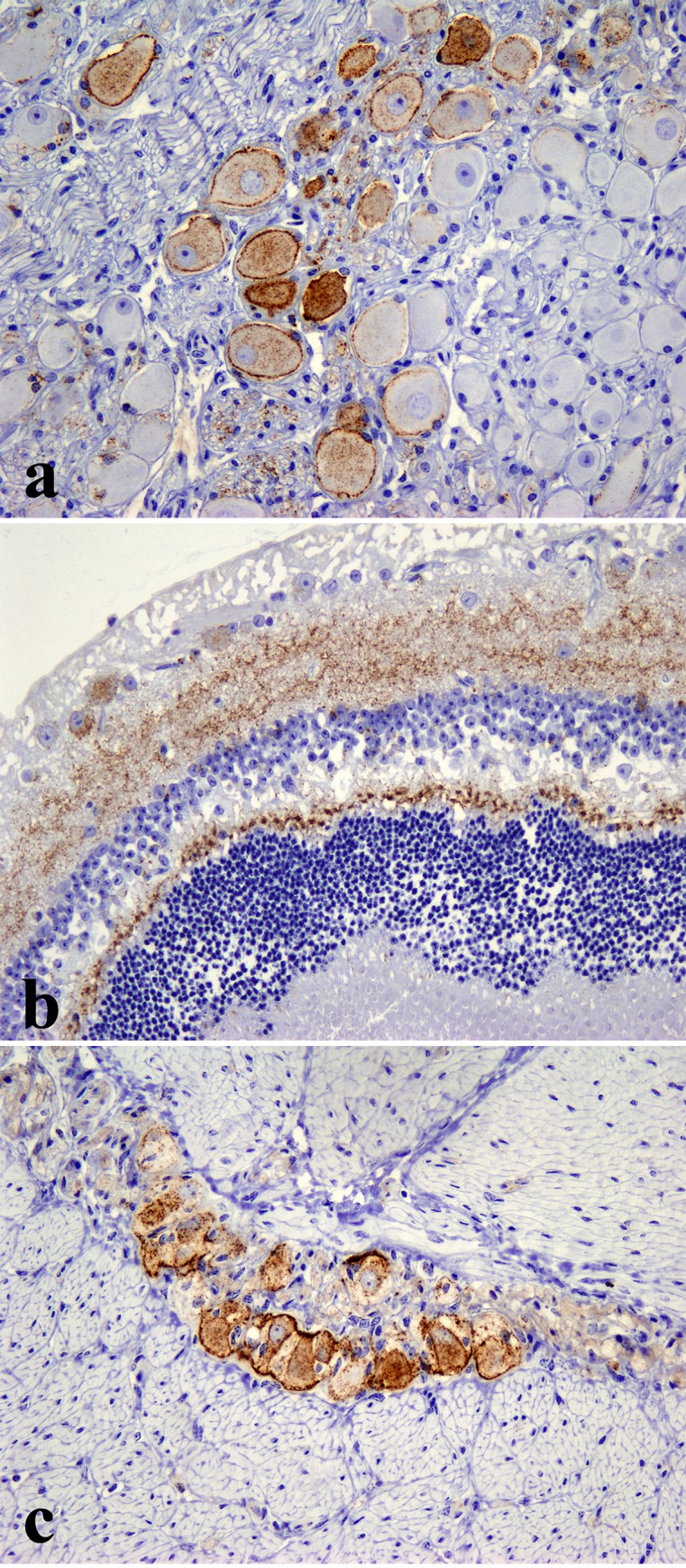
Accumulation of PrP^d^ (brown pigment) in the sympathetic chain ganglion cells (a), the plexiform layers of the retina (b) and myenteric plexus (c) of the single clinically affected deer (ID 009). IHC with antibody Bar224, x200.

**Figure 4:**
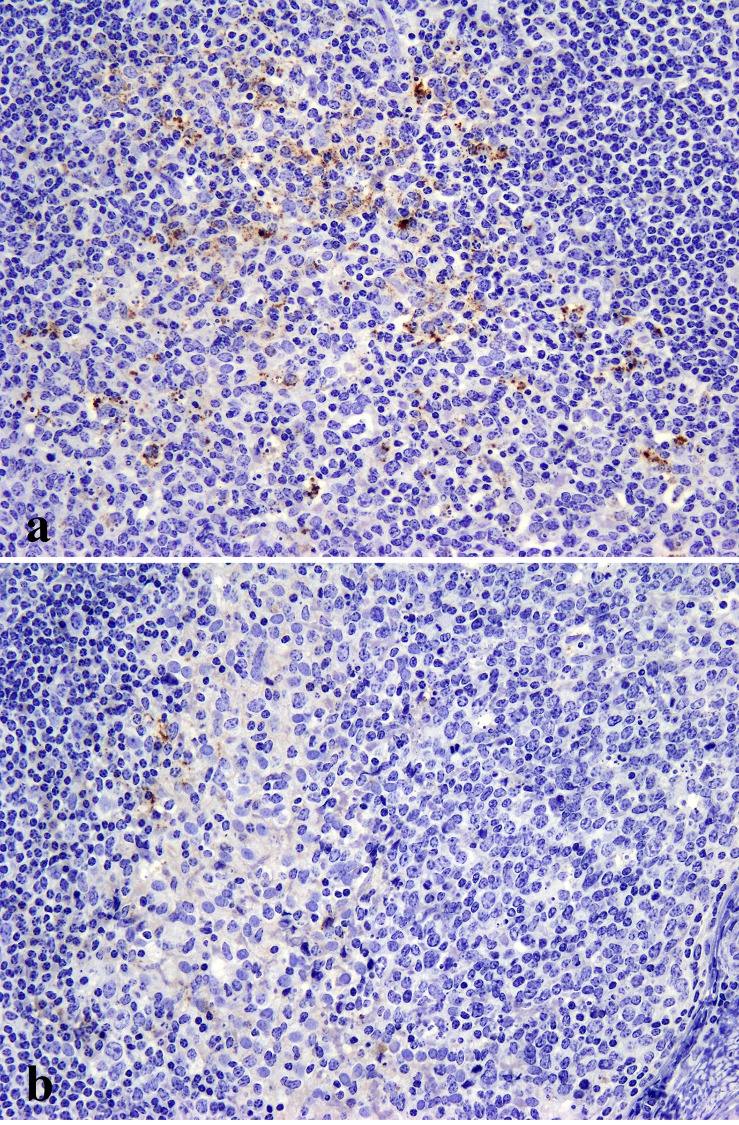
Accumulation of PrP^d^ (brown pigment) in tingible body macrophages present in the lymphoid follicles of the caecum (a) and colon (b) of the only clinically affected deer (ID 009). IHC with antibody Bar224, x200.

Immunohistochemically PrP^d^ was found in the clinically affected deer (ID 009) only and was largely restricted to the central and peripheral nervous systems (CNS and PNS respectively). Antibodies F99 and BAR224 were both equally effective at labelling PrP^d^ in deer tissue with no discernible differences. In the CNS, PrP^d^ accumulated predominantly in the brainstem and cerebellum with widespread diffuse particulate labelling of the neuropil and conspicuous intra-neuronal accumulation (Fig. 2). Peri-neuronal labelling was prominent in the dorsal motor nucleus of the vagus nerve (DMNV) and in the striatum. Intraneuronal granular accumulations were prominent in nuclei of the medulla, such as the accessory cuneate, spinal trigeminal (Fig. 2A) and posterior olivary nucleus (Fig. 2B), but were also present elsewhere in the brain with the exception of the Purkinje cells of the cerebellum. Intraneuronal PrP^d^ aggregates were greatly diminished or even absent when serial sections were incubated with the PrP N-terminal 12B2 antibody (Fig. 2C & 2D). Such a marked decrease in intracellular signal with preservation of the extracellular signal following labelling with PrP N-terminal specific antibodies is a consistent feature of BSE infections in several other species [5]. PrP^d^ was also detected in all segments of the spinal cord, in autonomic ganglia, cranial and peripheral nerves (Fig. 3A), sensory retina (Fig. 3B) and ganglion cells (myenteric plexus) throughout the enteric nervous system (Fig. 3C) which were frequently in close proximity to nearby lymphoid follicles, the majority of which were negative. However, sparse deposits of PrP^d^ were also detected within tingible body macrophages in a very small number of the lymphoid follicles within the caecum (Fig. 4A) and colon (Fig. 4B) but not in any other lymphoid tissue examined. All other organs examined including skin, cardiac and skeletal muscles, lung, liver, rumen, abomasum, small intestine, kidney, pancreas and mammary gland were negative for PrP^d^.

### Protein Misfolding Cyclic Amplification

**Figure 5:**
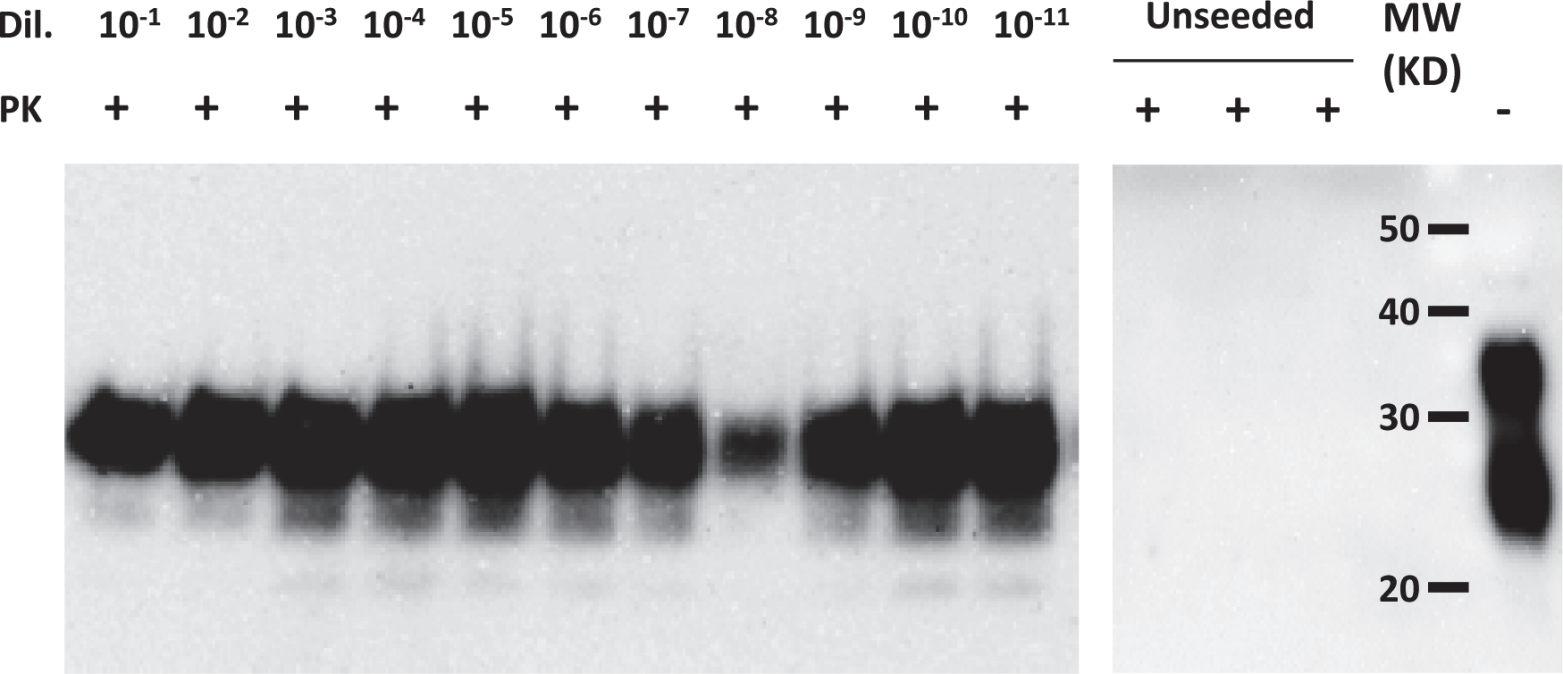
Western immnoblot for PrP^res^, using monoclonal antibody 2A11, of brain homogenate from initial titration experiment to determine the feasibility and sensitivity of serial protein misfolding cyclical amplification (sPMCA) using cattle brain homogenate for the detection of BSE agent in tissues from European red deer. Brain tissue from the single European red deer that developed clinical signs (009) used. Note the presence of PrP^res^ at a dilution of 10^−11^ after only three rounds of sPMCA showing exceptional sensitivity and complete lack of PrP^res^ in negative control samples showing lack of spontaneous conversion of PrP^C^ to PrP^res^. Dil = dilution, PK = proteinase-K treated (+) or not (−), MW = molecular weight markers.

The initial titration to determine the suitability of bovine brain homogenate for sPMCA with a European red deer brain sample (the single clinically positive animal, ID009) gave a positive result down to a sample dilution of 10^−11^ after 3 rounds of sPMCA (Fig. 5) confirming its suitability for use in this study.

**Figure 6:**
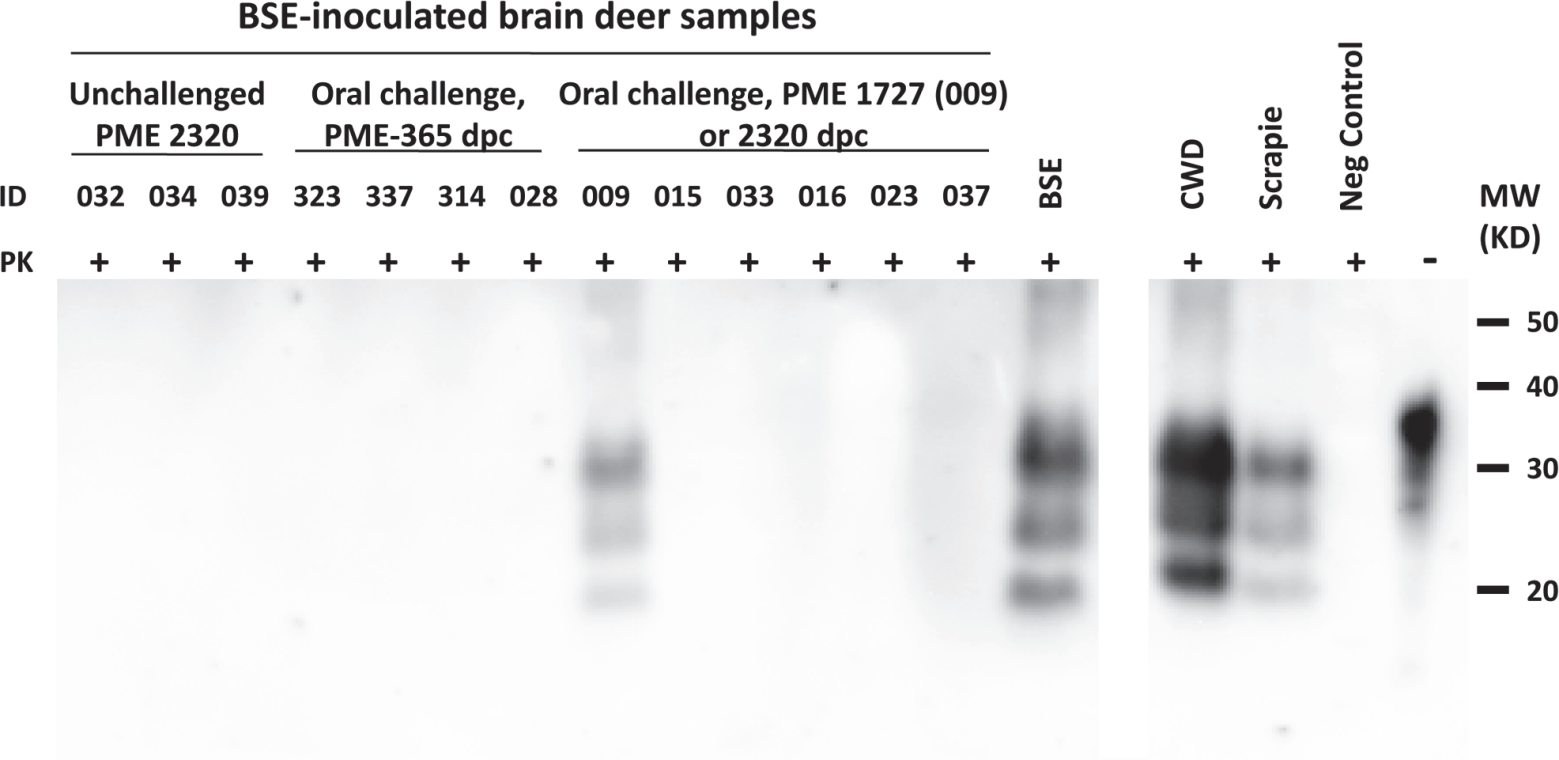
Western immunoblot for PrP^res^, using monoclonal antibody 2A11, of brain homogenates from orally challenged European red deer (n = 10) and unchallenged negative controls (n = 3) subjected to serial protein misfolding cyclical amplification (sPMCA), experiment two, round four. Note PrP^res^ is present only in brain homogenate from deer 009, the only animal that developed clinical signs which appeared at 1726 days after oral challenge with BSE, and the positive control samples (BSE positive bovine, scrapie positive ovine and chronic wasting disease (CWD) positive cervine brain homogenates). PME = post-mortem examination, dpc = days post-challenge, Neg Control = negative control, ID = deer identification number, PK = proteinase-K treated (+) or not (−), MW = molecular weight markers, KD = kilo Daltons.

All European red deer brain samples for both independant experiments of sPMCA from the single clinically positive animal (ID 009) were consistently positive for PrP^res^ in quadruplicate at all rounds examined (third round in the first experiment and third and fourth rounds in the second experiment, Table 1 and Fig. 6). All the rest of the samples were negative except a single tube of one of the quadruplicate samples in the third round from experiment one from a negative control animal (039) but which was negative in all rounds throughout experiment two (see Table 1).

## Discussion

This investigation resulted in the first and only known case, to date, of clinical disease or accumulation of abnormal PrP^d^ in any cervid species due to oral challenge with BSE. The increase in incubation period compared to European red deer challenged with BSE intra-cerebrally (1060 days) [33] compared to oral challenge (1727 days) is approximately 60% and similar to the differences observed in incubation periods for sheep or goats when challenged with TSE agents by these two routes [40,41]. The neurological clinical signs observed could be broadly related to the spongiform encephalopathy and the accumulation of PrP^d^ in that the restlessness, stereotypic head movements and pacing may be due to compromise of the nucleus accumbens [42], found in the striatum, and the laboured breathing due to the lesions in the medulla, where the respiratory centre is located [43]. Alternatively, the laboured and audible mouth breathing may have been due to, or contributed to by, compromise of either of the recurrent laryngeal nerves resulting in some degree of laryngeal paralysis but we were unable to determine this. Apart from the gradual loss of body weight, the speed of onset of clinical signs and progression was very rapid but animal welfare requirements precluded any further longitudinal study of these. The clinical signs described for this animal are broadly similar to those reported for clinical BSE in European red deer challenged via the intracerebral route [33], clinical cases of CWD in deer [44] and clinical cases of BSE in cattle [45].

The predominant hindbrain vacuolation pattern in this clinically affected European red deer challenged orally with BSE was similar to the same species challenged intra-cerebrally with the same BSE inoculum [33], cattle naturally infected with BSE [46], sheep challenged orally with the BSE agent [41] and deer and elk naturally infected with CWD [47]. Additionally, the tissue distribution of PrP^d^ in the clinically affected European red deer, which was restricted primarily to the central and peripheral nervous systems, was similar to that seen in the same species challenged intracerebrally with the same BSE inoculum [33]. However, this was in contrast to deer and elk naturally infected with CWD [44] and sheep naturally [48] or experimentally challenged via the oral route with scrapie [5] or BSE [41] which all have extensive peripheral accumulation of PrP^d^ in the lymphoid tissues and supports the body of work that states that both the host species and the TSE agent both play roles in determining the pattern of accumulation of PrP^d^ [4,5]. The PrP^d^ labelling in the brain of the clinically affected European red deer was predominantly punctate and granular and this pattern, along with the sites listed, is typical of accumulations in the brains of other ruminant sources of BSE infection [41].

The presence of PrP^d^ in a small number of tingible body macrophages in gut-associated lymphoid follicles in the clinically affected European red deer was not present when the same species was challenged by the intracerebral route with the same inoculum [33]. The mechanism of spread of accumulation of PrP^d^ in the intracerebrally challenged European red deer was probably incubation-period related centrifugal spread from the brain to the spinal column, cranial and other peripheral nerves [33]. This is thought also to be the major mechanism of dissemination in cattle infected by the oral route with the BSE agent after initial haematogenous spread from the intestine to the brain [49]. Studies have examined, in detail, the anatomy of initial translocation of both the BSE [50] and scrapie [10] agents across the intestinal mucosal barrier of sheep and concluded that the villous lacteals of the lamina propria and submucosal lymphatics are the key route in the first two to three and a half hours of exposure. PrP^d^ was not found in draining lymph nodes until 24 hours post-challenge and not before 30 days in Peyer’s patches [10] suggesting that the lymph node accumulation was probably residual inoculum and that in the Peyer’s patches was probably *de novo* PrP^d^. As PrP^d^ was detected only in the lymphoid tissue of the clinically affected European red deer challenged orally with BSE after an incubation period of 1727 days, and then only in very small amounts, and not those examined at 180 and 360 dpc nor those challenged intracerebrally which were sampled at 794–1290 dpc [33] it would appear that this is *de novo* PrP^d^ rather than residual inoculum. This being the case, European red deer challenged orally with BSE appear very similar to cattle with respect to organ distribution of PrP^d^ [51,52]. These findings further support the belief that the accumulation and distribution of PrP^d^ are influenced by a combination of the TSE agent and the host species [4,5].

The only animal to develop clinical disease in this study was homozygous for glutamine at *PRNP* codon 226 and, unfortunately, this was the only animal of this genotype in the group allowed to progress to clinical disease or termination of the experiment (Table 1). At the commencement of this study the only known genetic variation in the *PRNP* of cervid species closely related to European red deer and known to influence TSE susceptibility was codon 132 in elk where heterozygosity (methionine/leucine) was thought to confer a degree of resistance/lengthening of the incubation period in CWD infections [25–27]. This was supported by codon 132 in elk being the equivalent of human *PRNP* codon 129 which is highly influential in susceptibility to both sporadic and variant CJD [18,53]. Therefore, the European red deer in this study were initially subjected to evaluation of *PRNP* codon 132 only and as all were homozygous for methionine they were randomly assigned to the four experimental groups (6 month cull, 12 month cull, progression to clinical signs/termination of experiment and unchallenged negative control). At a later date it was shown that codon 226 of deer *PRNP* contained a polymorphism [28] that may be associated with TSE susceptibility [29] hence this was determined retrospectively after the animals had been assigned to their groups and already challenged with the BSE agent. Although it is tempting to speculate that European red deer homozygous for glutamine at *PRNP* codon 226 may be genetically more susceptible to BSE by the oral route than deer carrying glutamate at codon 226, one clinical animal cannot be statistically sufficient to make this association and further trials are required. Similar problems have been encountered in other long-term TSE studies where variations in previously unexamined *PRNP* codons have subsequently been shown to significantly affect the clinical disease outcome [11]. The only published assessments of the frequency of the various genotypes of *PRNP* codon 226 for European red deer were from those in Scotland were the QQ genotype was found in only 12.9% of animals over the whole country compared to 50% which were EE and 37.1% EQ [28]. However, one well studied island population within Scotland, which has no exchange with the mainland, had a prevalence for the QQ genotype of only 6.0% and the whole study examined 132 animals only [28]. If this study is representative of the European red deer population then the prevalence of the *PRNP* codon 226 QQ genotype is the lowest.

The presence of possible infectivity in both the single clinically affected animal and the non-clinically affected animals would have ideally been assessed by subjecting the resultant European red deer brain material to *in vivo* infectivity studies in a rodent model of shorter incubation period and/or further passage in European red deer but this was out-with the scope of this study. Fortunately, sPMCA was available and due to its exceptionally high sensitivity it has been proposed as a credible replacement for bioassay [54]. Our initial trials using bovine brain tissue as a substrate for detecting BSE by sPMCA showed that it was highly suitable for testing tissue from European red deer being able to detect a positive result to a sample dilution of 10^−11^ and, therefore, would probably be suitable for testing other closely related deer species, if not all cervid species. The consistent presence of PrP^res^ in 4/4 tubes at all rounds in both experiments of sPMCA is strongly suggestive that infectivity was present in the single clinically affected European red deer. The single positive tube, one of four replicates, from a negative control animal (039) in round three of the first experiment of sPMCA was considered negative and to be due to contamination of the sample at some point as all subsequent rounds from this animal in experiment two were negative for PrP^res^. Additionally, as all replicates at all rounds of the unseeded BSE negative bovine brain homogenate were negative it is unlikely this was a stochastic event.

The results of this study show that alimentary transmission of BSE to European red deer is possible but the transmission rate is low. However, culling of red deer in the UK, particularly males, occurs mostly when they are 8–10 years (2920–3650 days) old and therefore well within the possible incubation period for BSE [55]. Altogether our data suggest that when deer carcases for human consumption are subjected to the same regulations as other ruminant carcases (The Specified Bovine Offal Order 1990 [56] and its amendments) the zoonotic risk of BSE from consuming muscle from European red deer is probably very low.
